# Left-Right Function of *dmrt2* Genes Is Not Conserved between Zebrafish and Mouse

**DOI:** 10.1371/journal.pone.0014438

**Published:** 2010-12-28

**Authors:** Raquel Lourenço, Susana S. Lopes, Leonor Saúde

**Affiliations:** 1 Instituto de Medicina Molecular e Instituto de Histologia e Biologia do Desenvolvimento, Faculdade de Medicina da Universidade de Lisboa, Lisboa, Portugal; 2 Instituto Gulbenkian de Ciência, Oeiras, Portugal; New Mexico State University, United States of America

## Abstract

**Background:**

Members of the Dmrt family, generally associated with sex determination, were shown to be involved in several other functions during embryonic development. Dmrt2 has been studied in the context of zebrafish development where, due to a duplication event, two paralog genes *dmrt2a* and *dmrt2b* are present. Both zebrafish *dmrt2a/terra* and *dmrt2b* are important to regulate left-right patterning in the lateral plate mesoderm. In addition, *dmrt2a/terra* is necessary for symmetric somite formation while *dmrt2b* regulates somite differentiation impacting on slow muscle development. One *dmrt2* gene is also expressed in the mouse embryo, where it is necessary for somite differentiation but with an impact on axial skeleton development. However, nothing was known about its role during left-right patterning in the lateral plate mesoderm or in the symmetric synchronization of somite formation.

**Methodology/Principal Findings:**

Using a *dmrt2* mutant mouse line, we show that this gene is not involved in symmetric somite formation and does not regulate the laterality pathway that controls left-right asymmetric organ positioning. We reveal that *dmrt2a/terra* is present in the zebrafish laterality organ, the Kupffer's vesicle, while its homologue is excluded from the mouse equivalent structure, the node. On the basis of evolutionary sub-functionalization and neo-functionalization theories we discuss this absence of functional conservation.

**Conclusions/Significance:**

Our results show that the role of *dmrt2* gene is not conserved during zebrafish and mouse embryonic development.

## Introduction

The organization of the axial skeleton and skeletal muscles is bilaterally symmetric. In contrast, vertebrates are also characterized by stereotypic LR asymmetries in the distribution of the internal organs such as the heart and stomach on the left, and the liver on the right [1].

The axial skeleton and skeletal muscles are derived from embryonic structures called the somites. The epithelialization of a new pair of somites occurs in a bilateral symmetric manner from the anterior-most region of the mesenchymal presomitic mesoderm (PSM) [2]. This process is tightly regulated in space and time, with a new pair of somites of approximately the same size being formed with a regular species-specific time period [2].

The “clock and wavefront” model [3] postulates the existence of two independent phenomena accounting for periodic somite formation. The clock is evident in the PSM as periodic oscillations in gene expression of the so-called cyclic genes. These genes show a dynamic and reiterated expression in PSM cells with the same periodicity of somite formation [2]. Although the list of cycling genes is increasing, the conserved ones across species include mainly Notch targets, namely the bHLH (basic-helix-loop-helix) transcription repressors, the *hes* genes in the mouse and the *her* genes in zebrafish. More recently, a large scale transcriptome analysis revealed that the segmentation clock mechanism shows different degrees of complexity between mouse and zebrafish. In the mouse, many of the cyclic genes belong not only to the Notch pathway but also to the Wnt and FGF pathways [4]. In zebrafish there is no evidence for the existence of cyclic genes of the Wnt or FGF pathways [2]. In addition to the presence of a molecular clock, the PSM cells are under the influence of a wavefront of differentitaion. This wavefront is determined by gradients of Fgf and Wnt signalling coming from the posterior region of the embryo and fading towards the anterior portion of the PSM. While under the influence of Fgf/Wnt signalling, the PSM cells are maintained in an immature state and are prevented from starting the genetic program of somite formation [5], [6]. Soon after being formed the somites differentiate into the dermomyotome, which segregates into the dermal layer of the skin and skeletal muscles, and into the sclerotome that forms the vertebral column [7].

At the same time somites are being formed, left-right asymmetric information is establishing laterality in the nearby lateral plate mesoderm (LPM), culminating with the asymmetric positioning of internal organs. Before there are any signs of asymmetric organ localization in the vertebrate embryo, a conserved cascade of asymmetrically expressed genes is activated around the node in the mouse and around the Kupffer's vesicle (KV), the functionally equivalent fish organ. An excess of Nodal activity on the left side of the node/KV is transferred to the left LPM and in this location Nodal exerts a positive feedback on itself. As a consequence, the expression of nodal is amplified in the left LPM. Nodal also activates its negative regulators, the lefty genes. Lefty1 in the midline prevents *nodal* activation on the right LPM, while Lefty2 restricts the domain of *nodal* expression on the left LPM. The strong *nodal* expression on the left LPM induces *pitx2* expression that in turn activates morphogenetic proteins required for LR asymmetry of the internal organs [8]. Even though this Nodal cascade is conserved, the mechanism that induces nodal in the node/KV is different between vertebrates. Notch signaling activates *nodal* in the murine node region, while in zebrafish it activates the Nodal negative regulator *charon* around the KV [9], [10]–[12]. In addition to Notch signaling, Fgf8 and Wnt3a regulate *nodal* expression in the mouse node [13], [14]. The role of Fgf and Wnt signaling in controlling *nodal* expression at the KV has not been determined.

Somitogenesis and LR patterning share the same signalling pathways that occur at overlapping developmental time windows and in nearby embryonic tissues. For this reason, the asymmetric signals from the node have to be able to reach the LPM without affecting the bilateral symmetry of somite formation in the adjacent PSM. In fact, several lines of evidence show that bilateral symmetry is not a default state but instead has to be actively maintained through a mechanism that protects this territory from the LR asymmetric signals [15].

Retinoic acid (RA) has emerged as a conserved keeper of bilateral somite formation by buffering the PSM from the influence of LR cues [16]–[19]. Several lines of evidence show that Fgf8 and Snail1 are the LR cues that are being antagonized by RA signaling in the PSM [17], [19]–[22]. In zebrafish, another key player regulating development along the LR axis is the zinc-finger like transcription factor Dmrt2a/Terra, that belongs to the Dmrt (Doublesex (dsx) and Mab-3 Related Transcription Factor) family. We have previously shown that in zebrafish when Dmrt2a/Terra function is blocked, the expression of the cycling genes becomes desynchronized between the left and right sides and as a consequence somite formation is no longer symmetric. In addition, the positioning of the internal organs is compromised as a result of a randomization of left side LPM markers [23].

On the other hand, the mouse *dmrt2* null mutants have severe somite differentiation defects but nothing was known regarding a possible role of Dmrt2 in regulating symmetric somite formation and establishing the LR asymmetry pathway [24].

Here we report that *dmrt2* homozygous mouse mutants do not show LR desynchronization of somite formation and do not have LR defects regarding internal organs positioning. We show that *dmrt2a/terra* is expressed in the zebrafish KV in agreement with its function in LR development. In contrast, we did not detect *dmrt2* expression in the mouse node, consistent with its non-conserved function during the process of LR axis determination in this vertebrate.

## Results

### The murine Dmrt2 is not required for bilateral synchronization of somite formation

The process of somite formation is under the control of a molecular clock, revealed by the dynamic expression of the cyclic genes. This expression pattern is subdivided into three consecutive phases that are reiterated with the formation of each somite pair [2]. Importantly, the expression pattern of the cyclic genes on the left side of the PSM is always in phase with the one present on the right side.

In zebrafish, the expression of the cyclic genes *deltaC*, *her1* and *her7* becomes out of phase between the left and right sides of the PSM when *dmrt2a*/*terra* is knocked-down [23]. This eventually leads to the formation of an extra somite on either the left or the right side of the embryonic axis. Hence, it was proposed that Dmrt2a/Terra maintains the symmetry of somite formation possibly by protecting the PSM from the influence of LR asymmetric cues. This protection is only needed in a specific developmental time window that corresponds to the timing of transfer of LR information to the LPM [23]. In the mouse, it was shown that Dmrt2 is required for somite differentiation with severe implications on axial skeleton development [24]. However, its potential role in synchronizing the molecular clock that underlies somite formation was not evaluated in murine embryos.

**Figure 1:**
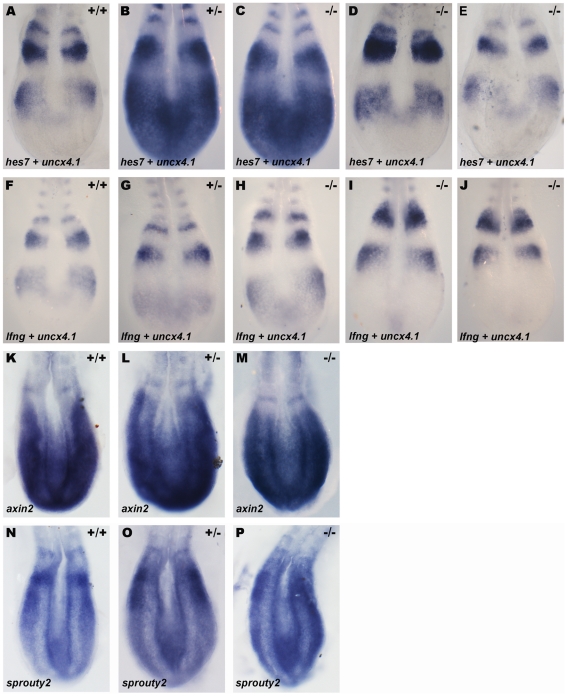
Dmrt2 is not required for symmetric somite formation in the mouse embryo. (A–P) Expression pattern of the cyclic genes *hes7* (A–E), *lfng* (F–J), *axin2* (K–M) and *sprouty2* (N–P) in E8.5 mouse embryos. (A–J) Embryos were also hybridized with the somite marker *uncx4*.*1*. (A, F, K, N) WT, (B, G, L, O) heterozygous and (C–E, H–J, M, P) *dmrt2* homozygous mutant embryos show the same phase of *hes1*, *hes7*, *axin2* and *sprouty2* cyclic expression in the PSM. All views are dorsal with anterior to the top.

In order to investigate the effect of abolishing Dmrt2 function in the mouse molecular clock, we analyzed the expression of cyclic genes of the Notch, Wnt and Fgf pathways in the context of *dmrt2* null mutants [24]. Embryos from *dmrt2* heterozygous crosses were collected between 8 to 15-somite stages, the developmental window that corresponds to the time when the PSM needs to be protected from LR signals [17]. These embryos were genotyped by PCR and analyzed by whole-mount *in situ* hybridization to reveal the expression of the Notch pathway cyclic genes *hes7* and *lfng*, the Wnt pathway cyclic gene *axin2* and the Fgf pathway cyclic gene *sprouty2* [6], [25], [26], [27]. Similarly to their wild-type and heterozygous siblings, in *dmrt2* homozygous mutants the dynamic expression of *hes7* (n = 8) (fig. 1A–E) and *lnfg* (n = 14) (fig. 1F–J) was not affected and normal phases of cyclic gene expression were observed. In addition, in all embryos analyzed, cyclic gene expression was in the same phase and therefore bilaterally symmetric on the left and right PSM's (fig. 1A–J). When analyzing the expression of *axin2* and *sprouty2,* the same results were observed (fig. 1K–P). The expression of *axin2* (n = 3) (fig. 1K–M) and *sprouty2* (n = 3) (fig. 1N–P) in wild-type, heterozygous and *dmrt2* homozygous mutant embryos was not affected and normal phases of cyclic gene expression were bilaterally symmetric in the PSM. In clear contrast to its role in zebrafish, Dmrt2 is not necessary to maintain bilateral symmetry of cyclic gene expression and therefore is dispensable for symmetric somite formation in the mouse embryo.

### Mouse left-right LPM patterning is independent of Dmrt2 function

The distribution of the internal organs is controlled by a conserved LR patterning cascade of information that starts early in development in the node/KV region and is then transferred asymmetrically to the LPM.

In zebrafish, Dmrt2a/Terra is necessary for the establishment of LR asymmetries. In the absence of Dmrt2a/Terra, the transfer of the *nodal*-related gene *spaw*, from the KV region to the left LPM, is randomized instead of being consistently transferred to the left LPM. In addition, the expression of the *spaw* downstream target, *pitx2*, is also randomized in the LPM. Concomitant with this randomization of left-side specific genes, heart positioning is also misplaced [23].

**Figure 2:**
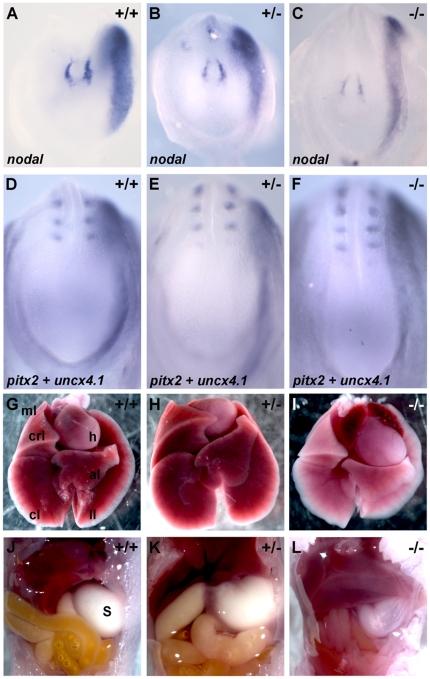
Dmrt2 is not required for the left-right LPM patterning in the mouse embryo. (A–F) Embryos between E8.0 and E8.5 processed by whole mount *in situ* hybridization with *nodal* (A–C) and *pitx2* (D–F) probes, respectively. (A–C) *nodal* expression is restricted to the left side of the node and left LPM in (A) WT, (B) heterozygous and (C) *dmrt2* homozygous mutant embryos. (D–F) *pitx2* expression is also restricted to the left LPM in (D) WT, (E) heterozygous and (F) *dmrt2* homozygous mutant embryos. (G–L) Localization of the internal organs in newborn mice. (G) In WT, (H) heterozygous and (I) *dmrt2* homozygous mutants the heart always bend to the left, the left lung always has one lobe (ll) and the right lung has four lobes (ml, crl, cl, al). (J–L) Concomitant with the normal situs of the heart and lungs, the stomach is also always placed on the correct left side of the axis. ml (middle lobe); crl (cranial lobe); cl (caudal lobe); al (accessory lobe); ll (left lobe); h (heart); s (stomach). All views are ventral with anterior to the top.

To further evaluate the putative role of Dmrt2 in LR asymmetry patterning in mouse development, we studied the expression of left-sided specific markers in *dmrt2* null mutants [24]. Embryos from *dmrt2* heterozygous crosses were collected between E8.0 and E8.5, genotyped and processed by whole-mount *in situ* hybridization to reveal the expression of the left LPM genes *nodal* and *pitx2* [28], [29]. At embryonic day E8.0, *nodal* expression was stronger on the left side of the node and restricted to the left LPM in both *dmrt2^−/−^* mutants (n = 8) and their siblings (fig. 2A–C). At embryonic day E8.5, in both *dmrt2* mutants (n = 9) and their sibling embryos, *pitx2* was expressed on the left side of the LPM (fig. 2D–F). This analysis reveals that Dmrt2 is not important to establish LPM laterality in the mouse embryo.

We next examined the laterality of the internal organs in newborn *dmrt2* mutants. Similarly to their siblings, *dmrt2* mutant mice (n = 16) showed normal internal organ organization (fig. 2G–L). The left lung had only one lobule, the right lung four lobules and the stomach and heart were always on the left side of the body cavities (fig. 2I, L). This data on organ localization is in agreement with the normal expression pattern of early LR markers and indicates that the role of Dmrt2 in establishing LR asymmetries is not conserved between zebrafish and mouse.

### *dmrt2a/terra* is expressed in the zebrafish Kupffer's vesicle but its homologous gene is absent from the mouse node

Our data clearly shows that the role of Dmrt2a/Terra in synchronizing somite formation and establishing LR asymmetries in the LPM, previously described in zebrafish [23], is not conserved in the mouse. We reasoned that this non-conserved role could be due to a differential pattern of expression in these two vertebrates.

**Figure 3:**
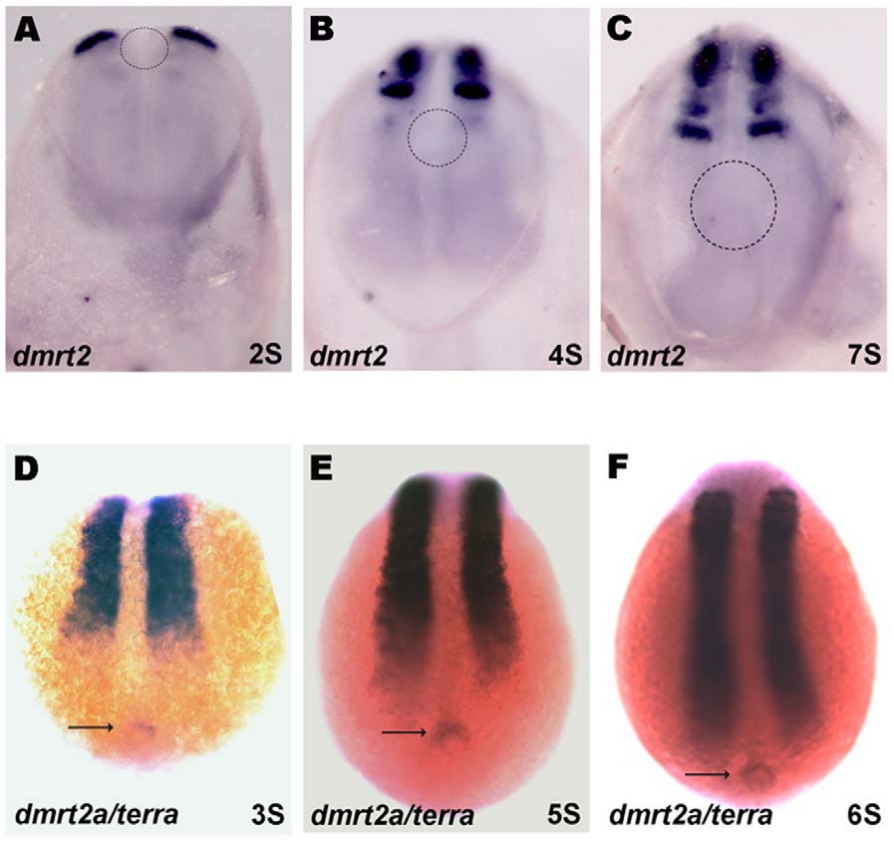
*dmrt2/terra* is expressed in the zebrafish kupfer's vesicle. (A–F) Whole mount *in situ* hybridization confirming the *dmrt2* conserved expression pattern in the anterior PSM and somites in mice (A–C) and zebrafish (D–F) embryos. Despite not being expressed in the mouse node (dashed circle) (A–C), *dmrt2a/terra* is present in the zebrafish equivalent structure, the kupffer's vesicle (arrow) (D–F). (A–C) Ventral views of mice embryos with anterior to the top. (D–F) Dorsal views of zebrafish embryos with anterior to the top. S, somite stage.

We confirmed that zebrafish *dmrt2a*/*terra* and its mouse homologue are expressed in the anterior region of the PSM and somites in both vertebrates (fig. 3A–F) [24], [30]. In addition, we could detect *dmrt2a/terra* transcripts in the zebrafish KV from the 3-somite stage until the 10-somite stage (fig. 3D–F). However, we could not find any expression of *dmrt2* in the mouse node from 1- somite stage to 7-somite stage, which is the equivalent laterality organ of zebrafish KV (fig. 3A–C). The differential expression in the laterality organs of zebrafish and mouse may explain the different role that this transcription factor plays in controlling LR laterality and symmetric somite formation in these two vertebrates.

## Discussion

The *dmrt* genes belong to a large family of transcription factors. These genes are present in several metazoan phyla (arthropods, nematodes and vertebrates) and show low sequence conservation outside the common DM domain, even within species [31]. The number of *dmrt* orthologue genes varies in different phyla, suggesting multiple instances of independent gene duplication and/or loss throughout evolution. In the vertebrate lineage alone the number of dmrt genes varies widely: eight genes (*dmrt1-8*) in human and mouse, five genes (*dmrt1-5*) in fish, two genes (*dmrt1* and *dmrt4*) in Xenopus and three genes (*dmrt1-3*) in chick [31], [32].

Despite being mainly expressed in developing gonads and associated with sex differentiation, not all the vertebrate *dmrt* genes are associated exclusively with this function. So far, from the eight known *dmrt* genes, five of them have already been implicated in other developmental processes other than sex differentiation. *dmrt* genes have been detected in the central nervous system (*dmrt3* in mouse, chick and medaka; *dmrt4* in mouse, *Xenopus*, medaka and platyfish; *dmrt5* in mouse, platyfish and zebrafish; *dmrt6* in mouse), nasal placodes (*dmrt3* in mouse and chick; *dmrt4* in *Xenopus* and platyfish; *dmrt5* in platyfish) and in the somites (*dmrt2* in mouse, chick and fish; *dmrt3* in chick) [32]. It is clear that Dmrt family members present a wide variation in gene number and their expression pattern suggests distinct functions. The possible variation at the level of gene function implies that during evolution paralog genes may have been subjected to either a sub-functionalization, with the functions of the ancestral gene being segregated into a set of paralog genes, or a neo-functionalization, with one of the paralog genes acquiring a new function. A careful functional analysis should be done to fully understand the extension of the role of these genes. Additional studies on Dmrt genes from ancestor organisms would give insight on the origin and evolution of these genes.

The first *dmrt* gene suggested to have a role unrelated to sexual development was *dmrt2*. It was detected in zebrafish and mouse PSM and somites and was reported to be absent from gonadal tissues [24], [30], [33]. Indeed, male and female homozygous mouse mutant embryos are obtained with the same frequency [24]. Functional studies in zebrafish showed that Dmrt2a/Terra protects the bilateral symmetric somite formation from the influence of LR cues. Without Dmrt2a/Terra the expression of the cycling genes in the PSM becomes desynchronized and consequently somite formation is no longer synchronized between the left and right sides of the zebrafish axis [23]. In addition, Dmrt2a/Terra was shown to establish asymmetry in the LPM, being necessary to restrict left-specific genes in the left LPM and having an impact in the localization of the heart on the left side [23]. In the mouse, these early Dmrt2 roles were not analyzed and only a later function in somite differentiation was reported [24].

To investigate the degree of functional conservation of *dmrt2* in mice, we started by characterizing the expression of the PSM Notch-related cycling genes *her7* and *lfgn*. This analysis was restricted to a specific developmental time window, 6–13 somites, which corresponds to the period when LR information is being transferred from the node to the left LPM - the time when PSM must be protected from the influence of these signals. The cyclic expression pattern of *her7* and *lfgn* in *dmrt2* null mutants showed no differences between the left and right sides of the PSM and consequently somite formation proceeded in a bilateral symmetric way. In contrast to the zebrafish, where all the cyclic genes identified so far belong to the Notch pathway, in the mouse several PSM cyclic genes are Wnt and Fgf pathway components. Among those are *axin2* and *sprouty2*, which are negative feedback inhibitors of the Wnt and Fgf pathway, respectively [34]. Similarly to what happens with the Notch cyclic genes, no desynchronization of *axin2* and *sprouty2* expression was observed between the left and the right PSM in *dmrt2* null mutants. This is in agreement with the idea supported by experimental data and computational modeling that suggest that oscillations in the Notch, Wnt and Fgf pathways are coupled and integrated in one molecular clock [34]; [35]. These results reflect a lack of conservation of the role of Dmrt2 during mouse development in what concerns LR synchronization of somite formation. Regarding a possible conservation of Dmrt2 function in establishing the LR asymmetry pathway, we looked at the expression of left-specific genes. In mice embryos mutant for *dmrt2* the expression of *spaw* and *pitx2* was restricted to the left LPM and consistently we observed a correct LR disposition of all the internal organs. Once again, these results indicate that mouse Dmrt2 does not play a role in the establishment of the LR asymmetric cascade.

During evolution the process of gene duplication is one key driving force for gene functional innovation. The evolutionary significance of gene duplication is explained by the duplication-degeneration-complementation (DDC) model, which states that the probability of a duplicate gene to be preserved increases with the occurrence of degenerative mutations in its regulatory region [36]. Since genes may have several functions that are controlled by different regulatory regions, when a specific subset of the gene function is subjected to a degenerative mutation, it may lose this given function and gain a new one. This leads to the emergence of different gene family members that become expressed in different tissues and/or developmental stages, thus allowing gene preservation during evolution [36].

Teleost fish underwent a genome duplication that occurred during the evolution of ray-finned fish. Recently, it was reported that, due to this genome duplication event, zebrafish *dmrt2a/terra* has a paralog gene named *dmrt2b* [37]. Contrary to *dmrt2a/terra*, that is present in all vertebrates, *dmrt2b* duplication only exists in the fish genome (zebrafish, tilapia, takifugu, tetraodon, medaka and stickleback), probably due to the fish lineage specific genome duplication [38]. In clear contrast to *dmrt2a/terra*, the fish-specific duplicated *dmrt2b* contributes to Hedgehog pathway [37]. Due to this new function of *dmrt2b*, these two genes are not redundant and therefore one does not compensate for the loss of the other [37]. The fact that *dmrt2b* allows an effective response to Hedgehog signaling explains the impact on somite differentiation in particular on slow muscle development [37]. Since Shh regulates the establishment of LR asymmetries in zebrafish [39] through its role in the midline, it is most likely that the LPM LR phenotype seen in *dmrt2b* morphants results from an impaired Hedgehog signaling at this level. This possibility is reinforced by the fact that we could not detect any *dmrt2b* expression in the zebrafish KV (data not shown). In addition, the fact that *dmrt2b* morphants lack desynchronizations of cyclic gene expression in the PSM [37] also suggests that the LR phenotype in these embryos arises from a different source when compared to *dmrt2a* LR phenotype.

We show here that *dmrt2a/terra* is expressed in the zebrafish KV, which is consistent with its role in LPM LR asymmetry establishment. In addition, we have previously shown that *dmrt2/terra* is asymmetrically expressed on the left side of Hensen's node in chick embryos [23]. Although no functional studies were performed, this expression pattern is highly suggestive of a LR patterning function of *dmrt2/terra* during chick development. In contrast to the fish and chick, we show here that *dmrt2* is not expressed in the mouse node, which is in agreement with the absence of a LR phenotype in *dmrt2* mutant mice.

Given the fact that zebrafish *dmrt2a/terra* is expressed in the KV and its homologue in chick Hensen's node, we propose that its expression and consequently its function in LR patterning was lost in the mouse lineage. This difference in function observed in mouse could arise: 1) from mutations occurring in the dmrt2 enhancer responsible for the node expression. To test this hypothesis, it would be interesting to combine bioinformatics and transgenics production to identify the zebrafish KV dmrt2 enhancer and show that it is indeed absent from the mouse genomic sequence; 2) from the loss in the mouse of a protein(s) necessary to activate specifically the node enhancer. In this situation, a bioinformatics analysis would reveal that the node enhancer will be present in the mouse genomic sequence and a new search for putative binding sites for known regulators could then be performed.

In contrast to the differential *dmrt2* expression in the KV/node reported in fish, chick and mouse, the expression at the level of the PSM and somites is conserved across all vertebrates studied so far [23], [24], [37]. In mouse, Dmrt2 is required for somite differentiation, in particular for patterning the axial skeleton [24]. In the absence of *dmrt2*, extracellular matrix components levels in the dermomyotome are downregulated leading to disrupted differentiation of the myotome. Furthermore, signaling between myotome and the sclerotome is compromised culminating with rib and vertebral malformations and postnatal death due to respiratory problems [24]. Therefore, the expression of *dmrt2* in the anterior PSM and somites correlates with its function in somite differentiation, a role already well described in mouse but still to be addressed in chick and zebrafish [24]. In zebrafish, *dmrt2a/terra* is necessary for bilateral somite formation due to its ability to synchronize the segmentation clock between the left and right PSM [23]. Since LR synchronized cycling gene expression starts already in the posterior PSM, the desynchronization observed in the absence of *dmrt2a/terra* is not easily explained by its expression in the anterior PSM and somites. We propose that *dmrt2a/terra* is synchronizing the clock in the posterior most part of the PSM, through its function in the KV. In the developing zebrafish embryo, the KV is placed in close contact with the most posterior part of the PSM and therefore makes it an ideal location to protect the PSM from the asymmetric signals that emerge from this organ. Again, since *dmrt2* expression in the mouse is absent from the node, no desynchronizations of cyclic gene expression are observed in the *dmrt2* mutants.

While some *dmrt* ortholog genes show a conservation of their function in different vertebrates, some may have divergent functions. Here we report one of such cases illustrated by the mouse ortholog of the zebrafish *dmrt2*.

## Materials and Methods

### Ethics Statement

All experiments involving animals were approved by the Animal User and Ethical Committees at the Instituto Gulbenkian de Ciência and Instituto de Medicina Molecular, according with directives from Direcção Geral Veterinária (PORT 1005/92).

### Zebrafish line

AB wild-type zebrafish were crossed and embryos collected and kept at 28°C until the appropriate stage [40].

### Mice breeding and genotyping

Mice carrying the *dmrt2* null mutation where previously described [24] and obtained from David Zarkower's laboratory. Dmrt2 heterozygous mice were maintained on a C57BL/6 genetic background. DNA was extracted from the tail tip of adult mice and genotyped by PCR using Dmrt2 specific primers. Dmrt2 WT forward primer 5′-CTGGACCCGAGTACAGTTCC-3′, Dmrt2 WT reverse primer 5′-AATGGTGCGTTCAACTCAGG-3′, Dmrt2 mutant forward primer 5′- TGCGGAGGGCTGGATCTTAAGGAG-3′ and Dmrt2 mutant reverse primer 5′-AGGGGGTGGGGATTTGACACCATC-3′, which resulted in a 830 bp band and a 270 bp band for the WT and mutant alleles, respectively. Dmrt2 heterozygous mice were crossed and embryos collected at specific stages (8.0–9.0 dpc). Mutant embryos were identified by PCR on DNA isolated from the yolk sacs using the same primers as for the adult mice genotyping.

### Cloning of mouse and zebrafish *dmrt2a/terra* and *dmrt2b* genes

Mouse *dmrt2* cDNA (IMAGE: 1248080) was used to synthesize an antisense RNA probe, linearized with EcoRI and transcribed with T3. Total RNA was extracted from appropriate staged zebrafish embryos using TRIzol (Invitrogen) and cDNA was synthesized with MMLV-Reverse Transcriptase kit (Promega). *dmrt2a/terra* full length sequence was amplified by PCR with the following primer set: *dmrt2a/terra* forward primer 5′-ATGACGGATCTGTCCGGCACG-3′ and *dmrt2a/terra* reverse primer 5′-AGCAAGAAGCCTTACTGAGATTTCCG-3′. *dmrt2b* was amplified by PCR with the following primer set: *dmrt2b* forward primer 5′-TTTCTTCCCGCTGTCAGACC-3′ and *dmrt2b* reverse primer 5′-TTATCTCATGAGCAGTGCCTCG-3′. The *dmrt2a/terra* amplified DNA fragment was cloned in the pCS2+ vector and *dmrt2b* in the pGEM-T easy vector. To synthesize the antisense *dmrt2a/terra* and *dmrt2b* RNA probes, plasmids were linearized with ApaI and SpeI and transcribed with SP6 and T7, respectively.

### Whole-mount *in situ* hybridization

Mouse embryos were analyzed by whole-mount in situ hybridization (WISH) as previously described [41] using the digoxigenin (DIG) labeled antisense RNA probes for *dmrt2*, *hes7*, *lfng*, *axin2*, *sprouty2*, *nodal* and *pitx2*. Zebrafish embryos were analyzed by WISH as previously described [42] using the digoxigenin (DIG) labeled antisense RNA probes for dmrt2a/*terra* and *dmrt2b*. Embryos were photographed with a LEICA Z6 APO stereoscope coupled to a LEICA DFC490 camera.
